# Editorial: Frontiers in Cardiovascular Medicine: Rising Stars 2021

**DOI:** 10.3389/fcvm.2022.928981

**Published:** 2022-06-09

**Authors:** Lijun Wang, Gui-e Xu, Longlu Pan, Elena Aikawa, Masanori Aikawa, Junjie Xiao, Ngan F. Huang

**Affiliations:** ^1^Institute of Geriatrics (Shanghai University), Affiliated Nantong Hospital of Shanghai University (The Sixth People's Hospital of Nantong), School of Medicine, Shanghai University, Nantong, China; ^2^Cardiac Regeneration and Ageing Lab, Institute of Cardiovascular Sciences, Shanghai Engineering Research Center of Organ Repair, School of Life Science, Shanghai University, Shanghai, China; ^3^Division of Cardiovascular Medicine, Department of Medicine, Brigham and Women's Hospital, Harvard Medical School, Boston, MA, United States; ^4^Departments of Cardiothoracic Surgery and Chemical Engineering, Stanford University, Stanford, CA, United States; ^5^Center for Tissue Regeneration, Restoration, and Rehabilitation, Veterans Affairs Palo Alto Health Care System, Palo Alto, CA, United States

**Keywords:** editorial, cardiovascular medicine, rising stars, 2021, frontiers

This editorial features the collection of articles published in *Frontiers in Cardiovascular Medicine: Rising Stars 2021*. The goal of this Research Topic is to promote the high-quality work of researchers at the early stages of their careers. The publications in this collection were selected based on their advancement in cardiovascular medicine and the research potential of the investigators. Here we celebrate the achievements of early-career investigators who thrive in the increasing hardships of academic research, including the fierce competition to secure research grant funding, and the publish-or-perish lifestyle of research dissemination (1). These rising stars possess determination, diligence, brilliance, and persistence—the qualities needed to survive the attrition rate and find success. The collection also represents the diversity of our scientific community. The following are the articles in the *Frontiers in Cardiovascular Medicine: Rising Stars 2021* collection.

*Proinflammatory Matrix Metalloproteinase-1 Associates with Mitral Valve Leaflet Disruption Following Percutaneous Mitral Valvuloplasty*, by Passos et al.. Mitral leaflet tearing is a serious and life-threatening complication of percutaneous mitral valvuloplasty (PMV). This original report demonstrates that pro-inflammatory factors such as MMP-1 and IFN-γ positively correlate with each other within valvular tissue and contribute to the localized degradation of collagen. Understanding the underlying mechanisms responsible for leaflet integrity disruption not only advances the fundamental insights into complications of PMV, but also is important for developing treatment strategies to treat mitral valve disease.

*Mechanoregulation of Vascular Endothelial Growth Factor Receptor 2 in Angiogenesis*, by Miller and Sewell-Loftin. Angiogenesis is essential for many cardiovascular diseases (2). This review comprehensively summarized the impact of VEGFR2 mechanosignaling in endothelial cells and its role in vascular dysfunction. The paper highlights the future research directions in VEGFR2 mechanoregulation in angiogenesis and provides important implications for developing new therapeutic strategies to combat cardiovascular diseases.

*Computational Screening Strategy for Drug Repurposing Identified Niclosamide as Inhibitor of Vascular Calcification*, by Tanaka et al.. This study employs computational screening methodology and functional studies to investigate niclosamide as a vascular calcification inhibitor. Vascular calcification is closely associated with many major adverse cardiovascular events (3). This publication utilizes drug repositioning strategy to identify novel therapeutic applications of existing drugs, thereby reducing time and cost compared to the traditional *de novo* drug discovery (4). The findings in this study offer a potential therapeutic compound for cardiovascular calcification treatment.

*Use of Multi-modal Data and Machine Learning to Improve Cardiovascular Disease Care*, by Amal et al.. With the rapid development of digital health and machine learning technologies (5, 6), data fusion can integrate multiple sources of information to improve the prediction of health risk factors. This paper reviews the state-of-the-art research on the latest techniques in data fusion in the field of cardiovascular medicine. This review highlights the use of multi-modal data and machine learning to improve precise prediction and treatment of cardiovascular diseases.

*The RiboMaP Spectral Annotation Method Applied to Various ADP-Ribosylome Studies Including INF-*γ*-Stimulated Human Cells and Mouse Tissues*, by Singh et al.. ADP-ribosylation plays important roles in various human diseases, including inflammatory vascular diseases (7). RiboMaP is a novel mass spectral annotation strategy to facilitate identification and reporting of ADP-ribosyl peptides and proteins (8). The authors of this study took advantage of publicly available ADP-ribosylome data to illustrate recent advances in the field of ADPr proteomics in relation to inflammation and cardiovascular disease, thereby extending the initial focus on this pathway for cancer therapeutics.

*Connections for Matters of the Heart: Network Medicine in Cardiovascular Diseases*, by Sonawane et al.. Network medicine is a developing field with applications in human cardiovascular disorders (9). Molecular-bioinformatics combined with omics data and artificial intelligence can advance discoveries in complex disease mechanisms as well as drug design. This publication discusses the approach of network medicine and its application to cardiovascular research to understand the diversity of diseases, identify novel mechanisms and develop new therapies for precision medicine.

*Single Cell Transcriptomic Analysis Reveals Organ Specific Pericyte Markers and Identities*, by Baek et al.. Single-cell transcriptomics provide a powerful approach to build detailed cellular maps at single-cell resolution (10). Despite the important role of pericytes in cardiovascular system, the underlying mechanism of their involvement are not yet clearly understood. This study identifies specific pericyte markers among lung, heart, kidney and bladder using single cell transcriptomics, and reveals differentially expressed genes and functional relationships between mural cells. This article provides new insights into distinguishing organ specific pericyte from other mural cell populations.

## Author Contributions

NH, JX, EA, and MA are topic editors of this special issue and contributed to writing and revising of this editorial. LW, G-eX, and LP drafted the editorial. All authors contributed to the article and approved the submitted version.

## Funding

This work was supported by the grants from National Key Research and Development Project (2018YFE0113500 to JX), National Natural Science Foundation of China (82020108002 to JX), Innovation Program of Shanghai Municipal Education Commission (2017-01-07-00-09-E00042 to JX), the grant from Science and Technology Commission of Shanghai Municipality (21XD1421300 and 20DZ2255400 to JX), Natural Science Foundation of Shanghai (19ZR1474100 to LW), and the Dawn Program of Shanghai Education Commission (19SG34 to JX).

## Conflict of Interest

The authors declare that the research was conducted in the absence of any commercial or financial relationships that could be construed as a potential conflict of interest.

## Publisher's Note

All claims expressed in this article are solely those of the authors and do not necessarily represent those of their affiliated organizations, or those of the publisher, the editors and the reviewers. Any product that may be evaluated in this article, or claim that may be made by its manufacturer, is not guaranteed or endorsed by the publisher.
